# Lipopolysaccharide administration for a mouse model of cerebellar ataxia with neuroinflammation

**DOI:** 10.1038/s41598-020-70390-7

**Published:** 2020-08-07

**Authors:** Jungwan Hong, Dongyeong Yoon, Youngpyo Nam, Donggun Seo, Jong-Heon Kim, Min Sung Kim, Tae Yong Lee, Kyung Suk Kim, Pan-Woo Ko, Ho-Won Lee, Kyoungho Suk, Sang Ryong Kim

**Affiliations:** 1 Brain Science and Engineering Institute, Kyungpook National University, Daegu, 41566 Republic of Korea; 2 School of Life Sciences, BK21 Plus KNU Creative BioResearch Group, Kyungpook National University, Daegu, 41566 Republic of Korea; 3 Department of Pharmacology, Brain Science and Engineering Institute, BK21 Plus KNU Biomedical Convergence Program, School of Medicine, Kyungpook National University, Daegu, 41944 Republic of Korea; 4 Bioengineering Institute, Corestem Inc., Seoul, 13486 Republic of Korea; 5 Department of Neurology, School of Medicine, Kyungpook National University, Daegu, 41944 Republic of Korea

**Keywords:** Molecular biology, Neuroscience

## Abstract

Most cerebellar ataxias (CAs) are incurable neurological disorders, resulting in a lack of voluntary control by inflamed or damaged cerebellum. Although CA can be either directly or indirectly related to cerebellar inflammation, there is no suitable animal model of CA with neuroinflammation. In this study, we evaluated the utility of an intracerebellar injection of lipopolysaccharide (LPS) to generate an animal model of inflammatory CA. We observed that LPS administration induced the expression of pro-inflammatory molecules following activation of glial cells. In addition, the administration of LPS resulted in apoptotic Purkinje cell death and induced abnormal locomotor activities, such as impaired motor coordination and abnormal hindlimb clasping posture. Our results suggest that intracerebellar LPS administration in experimental animals may be useful for studying the inflammatory component of CA.

## Introduction

Cerebellar ataxias (CAs) are motor neuron diseases caused by pathological processes affecting the cerebellum or its associated pathways^1,2^. They are characterised by symptoms such as abnormal coordination of balance, movement and gait. Currently, most CAs are incurable, with few exceptions, and only symptom alleviation is possible^3^. CAs can be caused by genetic defects, sporadic neurodegenerative disorders, and acquired conditions (for example, infections, toxic reactions, alcohol, and vitamin deficiencies), but they can also arise for unknown reasons^1,3,4^. In particular, many reports have shown a close relationship between cerebellar inflammation and CA^1,3,5^. The activation of glial cells resulting in the production of pro-inflammatory molecules has been observed in many animal models of CA and is also evident in the cerebellums of patients with CA^6–9^. Furthermore, cerebellar inflammation has been shown to induce the loss of Purkinje cells, which results in cerebellar dysfunction^1,7^. Although the understanding of CA pathogenesis associated with genetic causes, cerebellar neurotoxicity, and physical damage has contributed to the development of some animal models of CA^1,3^, there is no suitable animal model to study CA following glial activation and neurotoxic cerebellar inflammation in vivo.

Neuroinflammation plays a crucial role in the pathogenesis and progression of neurodegenerative disorders such as Parkinson’s disease and Alzheimer’s disease^10^. As a potent stimulator of neuroinflammation, lipopolysaccharide (LPS) has been used to mediate neurodegenerative progression^11^. An accumulation of evidence has shown that LPS-induced neuroinflammation causes neuronal damage through activation of microglia and astrocytes, M1 microglial polarization and the release of pro-inflammatory mediators. For example, LPS injected into the substantia nigra induces microglia activation, which causes the degeneration of dopamine neurons that constitutes the pathological basis of Parkinson’s disease^12^. In addition, LPS causes an increase in amyloid beta, which results in demyelination and oligodendrocyte injury in mice brains, which in turn causes Alzheimer’s disease^13^.

In the present study, for developing an animal model of CA associated with cerebellar inflammation, we evaluated the utility of an intracerebellar injection of LPS for the induction of glial inflammation-mediated neurotoxic events in the mouse cerebellum.

## Results

### Glial activation by LPS administration in the mouse cerebellum

**Figure 1:**
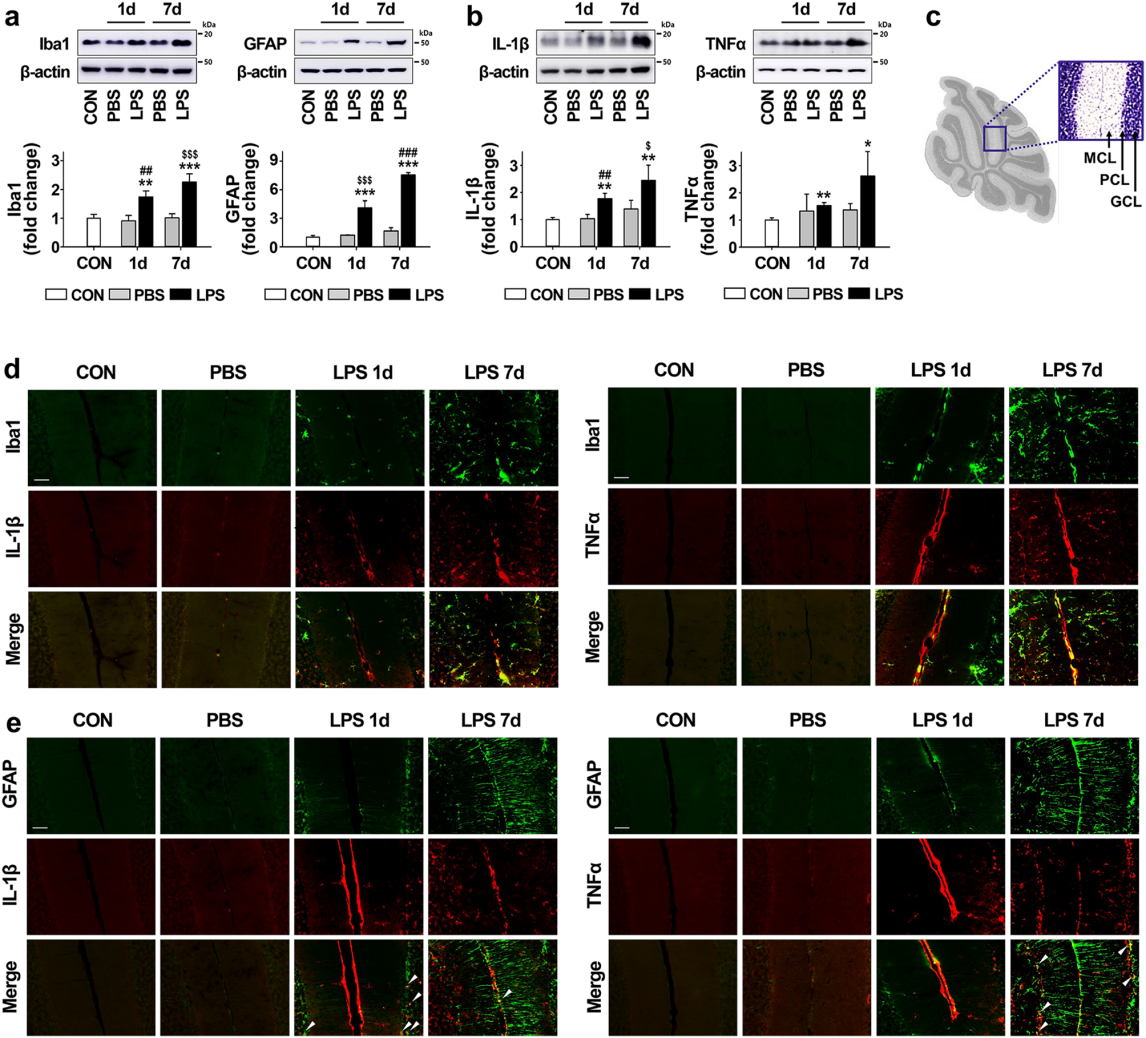
Inflammatory response increased by LPS injection in the cerebellum. (**a**) The expression levels of Iba1 (for microglia) and GFAP (for astrocytes) in the cerebellum 1 day and 7 days after LPS injection. Iba1: ^**^p = 0.008 and ^***^p < 0.001 versus CON, ^$$$^*p* < 0.001 versus PBS (7 days), ^##^*p* = 0.004 versus PBS (1 day) (n = 3 per group, *F*(4,10) = 26.219). GFAP: ^***^p < 0.001 versus CON, ^$$$^*p* < 0.001 versus PBS (1 day), ^###^*p* < 0.001 versus PBS (7 days) (n = 3 per group, *F*(4,10) = 150.249) (**b**) The expression levels of proinflammatory cytokines, such as IL-1β and TNFα, in the cerebellum 1 day and 7 days after LPS injection. IL-1β: ^**^p = 0.002 versus CON, ^$^*p* = 0.014 versus PBS (7 days), ^##^*p* = 0.003 versus PBS (1 day) (n = 3 per group, *F*(4,10) = 11.034). TNFα: ^**^p = 0.0027 and ^*^p = 0.035 versus CON (n = 3 per group, *t*(4) = − 6.632 and *t*(4) = − 3.136). (**c**) A sagittal histologic section of the mouse cerebellum. The box shows the cerebellar cortex about which the section shown in (**d**) and (**e**) was taken. We use “MCL” to represent the molecular cell layer, “PCL” to represent the Purkinje cell layer, and “GCL” to represent the granule cell layer. (**d**) The expression pattern between microglia (green) and proinflammatory cytokines (IL-1β and TNFα; red) in cerebellum 1 day and 7 days after LPS injection (Scale bar = 50 μm). (**e**) The expression patterns of astrocytes (green) and proinflammatory cytokines (IL-1β and TNFα; red) in the cerebellum at 1 day and 7 days after LPS injection. IL-1β and TNFα expression was significantly increased by activated microglia in the cerebellum after LPS injection compared to levels in the controls, but were rarely colocalized with astrocytes (arrowheads). Scale bar = 50 μm. All values are expressed as the mean ± standard deviation (SD). Statistical significance tested with one-way ANOVA with Tukey’s *post-hoc* analysis (**a** and **b**; IL-1β) and two-sided paired t-test (**b**; TNFα). The membranes were initially cut according to the molecular weight size of proteins. The images of blots were displayed in cropped format, and original blots of (**a**) and (**b**) with cropped demarcation of membranes are shown in Supplementary Fig. 2.

To develop an animal model of CA caused by inflammation, we injected LPS directly into the cerebellum as an effective initiator of neuroinflammation in this study. As shown by western blotting in Fig. 1a, the expression levels of Iba1 (for microglia) and GFAP (for astrocytes) were significantly increased by LPS injection in a time-dependent manner. Consistent with the expression results of Iba1 and GFAP, the expression levels of proinflammatory cytokines, such as IL-1β and TNFα, were significantly upregulated by 2.4 and 2.6 fold, at 7 days post-injection with LPS compared to intact controls (Fig. 1b). The levels of glia and proinflammatory cytokines in PBS-treated mice were not different from controls. Additionally, we performed double immunofluorescence staining of proinflammatory cytokines, IL-1β and TNFα, with cell-specific markers, Iba1 and GFAP, to identify the cell types that expressed the proinflammatory molecules after LPS treatment. The immunostaining results revealed that IL-1β and TNFα were distributed in the cerebellum following LPS treatment, mainly co-localized with Iba1-positive cells, and rarely co-localized with GFAP-positive cells (Fig. 1d,e, respectively), indicating that the proinflammatory cytokine induction by LPS predominantly occurred in microglia.

### Induction of chemoattractants by LPS administration in the mouse cerebellum

**Figure 2:**
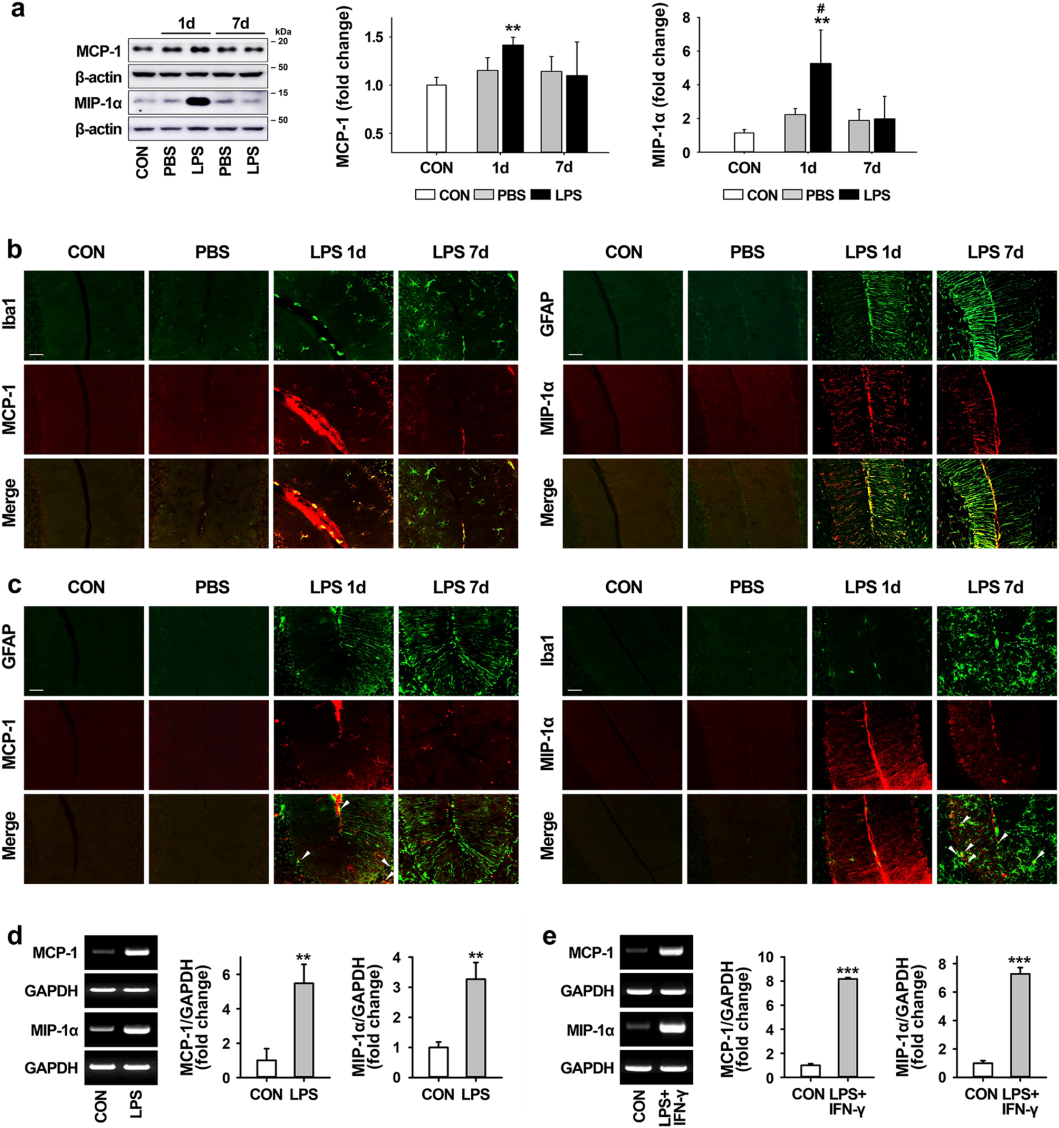
Induction of chemoatractants by LPS injection in the cerebellum. (**a**) The expression levels of proinflammatory chemokines, such as MCP-1 and MIP-1α, in the cerebellum at 1 day and 7 days post-LPS injection. MCP-1: ^**^p = 0.003 versus CON (n = 3 per group, *t*(4) =  − 6.26). MIP-1α: ^**^p = 0.008 versus CON, ^#^*p* = 0.049 versus PBS (1 day) (n = 3 per group, *F*(4,10) = 6.042). (**b** and **c**) The expression pattern between glial cells (microglia and astrocytes; green) and proinflammatory chemokines (MCP-1 and MIP-1α; red) in the cerebellum at 1 day and 7 days post-LPS injection (Scale bar = 50 μm). MCP-1 and MIP-1α expression were upregulated by microglia and astrocyte in the LPS-exposed cerebellum (**b**), but rarely colocalized with astrocytes and microglia, respectively (**c**, arrowheads). Scale bar = 50 μm. (**d** and **e**) Increases in mRNA expression levels of MCP-1 and MIP-1α by treatment with LPS and LPS + IFN-γ in primary microglia (**d**) and astrocyte cultures (**e**). Relative mRNA expression of MCP-1 and MIP-1α at 6 h after treatment with inflammatory stimuli was determined by RT-PCR. Graphs represent quantitative analysis of gel images normalized to GADPH. Microglia: ^**^p = 0.004 (MCP-1, *t*(4) =  − 6.003) and ^**^p = 0.003 (MIP-1α, *t*(4) =  − 6.750) versus CON (n = 3 per group). Astrocytes: ^***^p < 0.001 (MCP-1, *t*(4) =  − 74.306; MIP-1α, *t*(4) = − 22.787) versus CON (n = 3 per group). All values are expressed as the mean ± standard deviation (SD). Statistical significance tested with one-way ANOVA with Tukey’s *post-hoc* analysis (**a**; MIP-1α) and two-sided paired t-test (**a**; MCP-1, **d**, and **e**). The membranes were initially cut according to the molecular weight size of proteins. The images of blots were displayed in cropped format, and original blots of (**a**), (**d**) and (**e**) with cropped demarcation of membranes are shown in Supplementary Fig. 2.

We also examined the production of proinflammatory chemokines, such as MCP-1 and MIP-1α known as chemoattractants for inflammatory cells, in the LPS-injected cerebellum. As shown by western blotting (Fig. 2a), the expression of MCP-1 and MIP-1α in the cerebellum was significantly increased 1 day after LPS injection compared to levels in intact controls, and this increase abated at 7 days. Similar to the western blotting, the double immunostaining showed that the increases in these chemokines were more apparent at 1 day after LPS treatment than 7 days, and that the expression of MCP-1 was mainly observed in microglia, while MIP-1α was mainly observed in astrocytes (Fig. 2b), although some expression of each chemokine was also observed in both astrocytes and microglia in vivo (Fig. 2c), and the mRNA levels of both MCP-1 and MIP-1α (measured by traditional RT-PCR) increased in cultured primary microglia and astrocytes exposed to LPS and LPS + IFN-γ, respectively (Fig. 2d,e).

### Effects of LPS on microglial polarization in the mouse cerebellum

**Figure 3:**
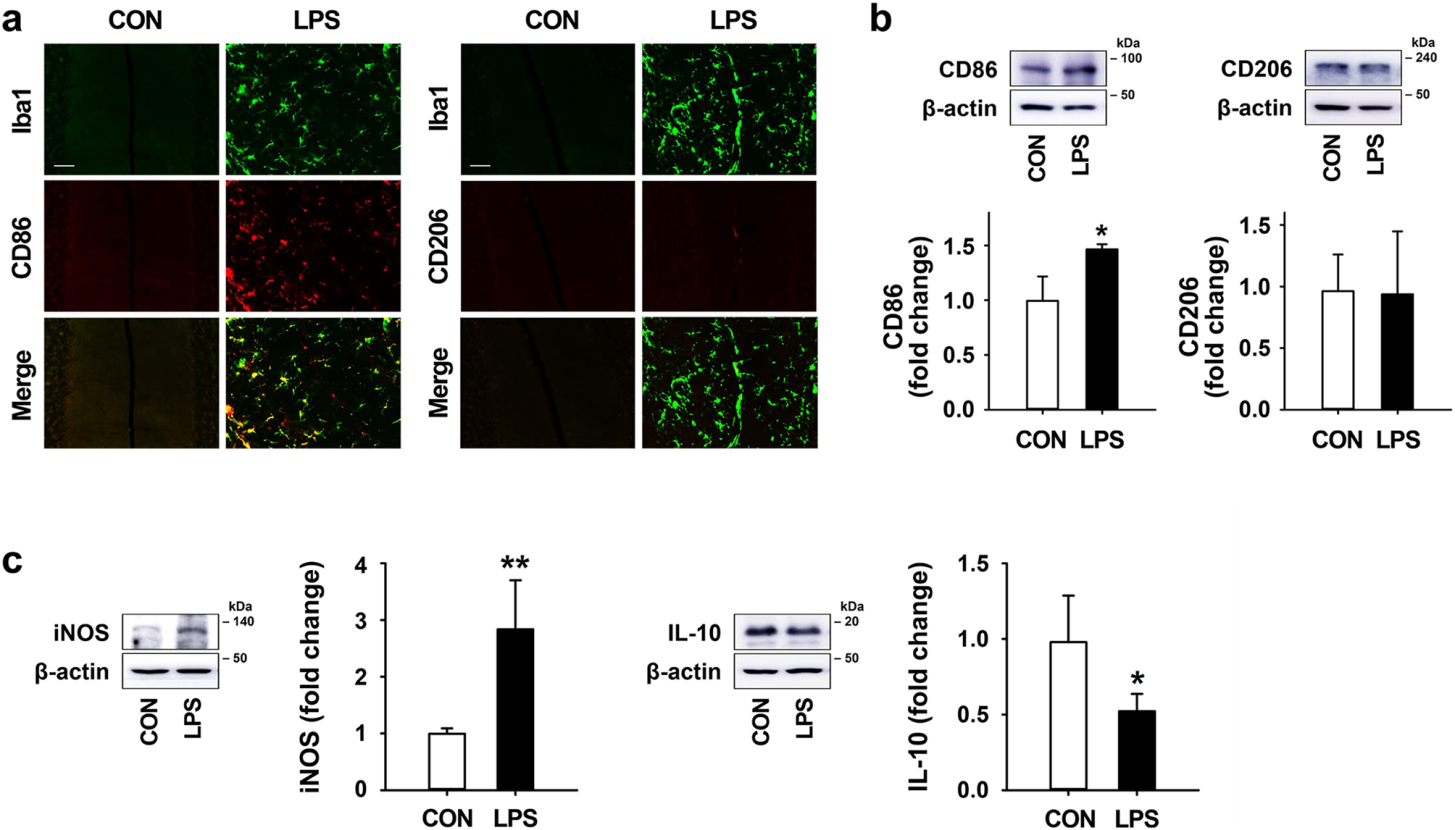
Effects of LPS on microglial polarization in the mouse cerebellum. (**a**) The expression pattern of microglia (Iba1; green) and M1/M2-type microglia marker (CD86 and CD206; red) in the cerebellum 7 days after LPS treatment (Scale bar = 50 μm). (**b**) The expression levels of CD86 and CD206 in the cerebellum at 7 days post-LPS injection. CD86: ^*^p = 0.038 versus CON, *F*(4,10) = 10.512). (**c**) The expression levels of iNOS (for M1 marker) and IL-10 (for M2 marker) in the cerebellum 7 days after LPS treatment. iNOS: ^**^p = 0.007 vs. CON (n = 4 per group, *F*(3,12) = 10.207). IL-10: ^*^p = 0.022 versus CON (n = 4 per group, *F*(3,12) = 25.728). All values are expressed as the mean ± standard deviation (SD). Statistical significance tested with one-way ANOVA with Tukey’s *post-hoc* analysis. The membranes were initially cut according to the molecular weight size of proteins. The images of blots were displayed in cropped format, and original blots of (**b**) and (**c**) with cropped demarcation of membranes are shown in Supplementary Fig. 2.

To examine the effects of LPS on the regulation of the microglial phenotype in the mouse cerebellum, we investigated the expression levels of M1- and M2-type microglia 7 days after LPS treatment. In the results of double immunostaining, CD86 (an M1 marker) was highly expressed in Iba1-positive microglia by LPS injection, whereas CD206 (an M2 marker) was rarely expressed in the cerebellum (Fig. 3a). Consistent with the double immunostaining results, western blotting showed that LPS exposure in the cerebellum induced a significant increase in CD86 compared to the level in intact controls with no change in CD206 expression (Fig. 3b). Moreover, in western blotting results, LPS treatment was found to increase the expression of iNOS (an M1 marker) and significantly reduce the expression of IL-10 (an M2 marker) relative to control levels (Fig. 3c).

### Motor impairments and Purkinje cell damage following to LPS-induced neuroinflammatory responses

**Figure 4:**
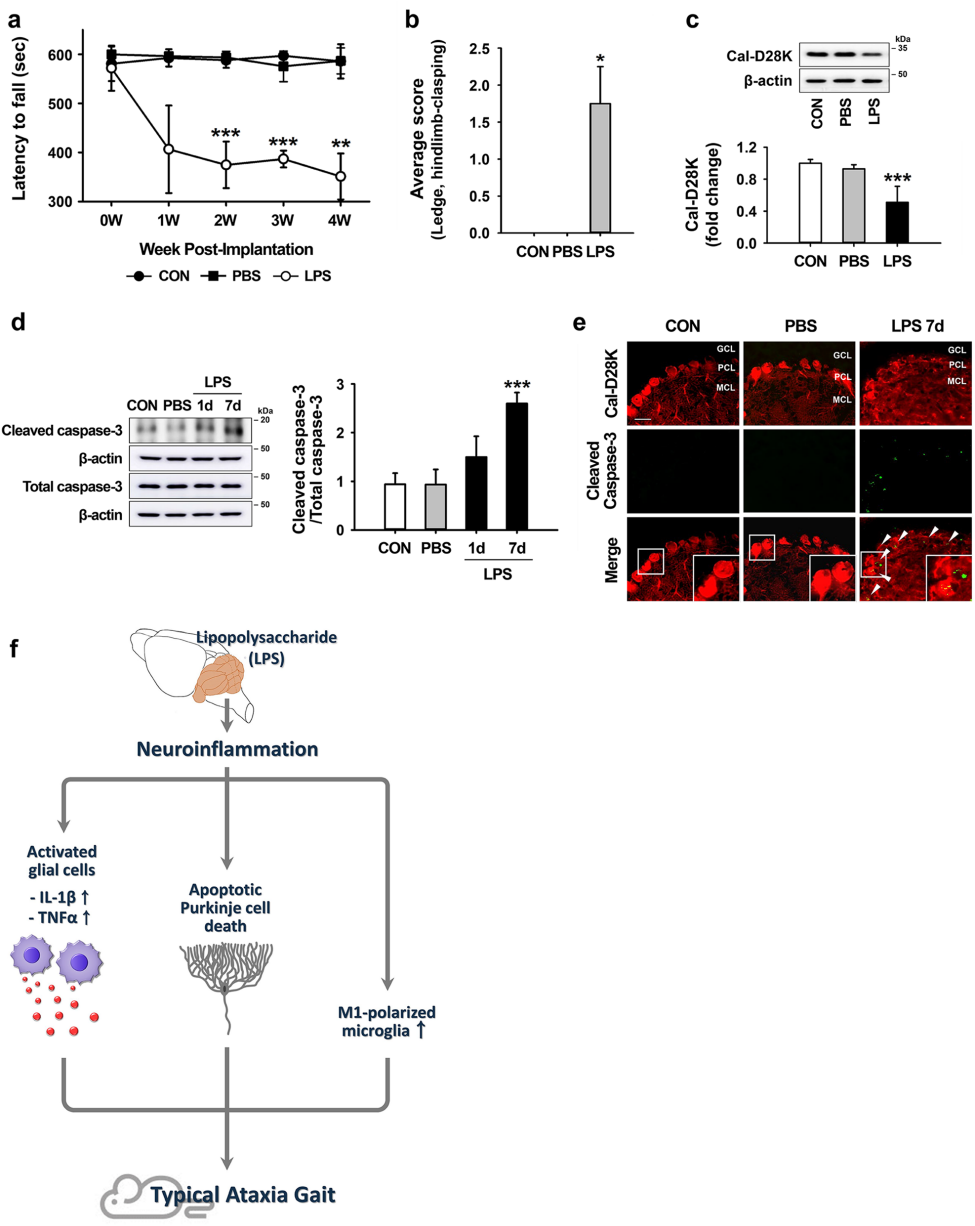
Behavioral impairment and apoptotic Purkinje cell death induced by inflammation in LPS-injected mice. (**a**) Rotarod test for 4 weeks after LPS injection. ^**^p = 0.001 and ^***^p < 0.001 versus CON (CON, n = 6; PBS, n = 4; LPS, n = 3; 2 W, *t*(5) = 8.316; 3 W, *t*(5) = 21.243; 4 W, *t*(5) = 6.690). (**b**) Simple composite phenotype scoring system at 4 weeks post-LPS injection. ^*^p = 0.029 versus CON (n = 4 per group). (**c**) The expression levels of Cal-D28K (for Purkinje cells) in the cerebellum 7 days after LPS injection. ^***^p < 0.001 versus CON (n = 4 per group, *F*(3,12) = 14.735) (**d**) The expression levels of activated caspase-3 in the cerebellum 7 days after LPS injection. ^***^p < 0.001 versus CON (n = 4 per group, *F*(3,12) = 25.842). All values are expressed as the mean ± standard deviation (SD). Statistical significance tested with two-sided paired t-test (**a**), Mann–Whitney U statistic (**b**), and one-way ANOVA with Tukey’s *post-hoc* analysis (**c** and **d**). (**e**) Detection of apoptotic Purkinje cells (arrowheads) in the cerebellum 7 days after LPS injection, using immunofluorescence analysis of cleaved caspase-3. We use “MCL” to represent the molecular cell layer, “PCL” to represent the Purkinje cell layer, and “GCL” to represent the granule cell layer. Insets delimited by the solid line in the panels on the right show high magnification portions of the image. (Scale bar = 100 μm). (**f**) Schematic representation of ataxia symptoms induced by neuroinflammation in the LPS-exposed cerebellum. The membranes were initially cut according to the molecular weight size of proteins. The images of blots were displayed in cropped format, and original blots of (**c**) and (**d**) with cropped demarcation of membranes are shown in Supplementary Fig. 2.

To investigate the suitability of the LPS-based animal model of inflammatory CA (ICA), we confirmed motor deficits in ICA mice through rotarod testing at weekly intervals for 4 weeks, beginning when the mice were 10-week-old. Retention time on the rotating rod significantly declined, from 572 ± 46 s at 0 week to 351 ± 47 at 4 weeks after LPS injection compared to the performance of intact controls (≥ 580 s) (Fig. 4a). Additionally, we carried out rapid and sensitive phenotype assessments for ataxia-like deficits 4 weeks after LPS injection, as previously described^14^. While there was no mortality in any of the groups (data not shown), LPS-injected mice showed impaired motor coordination (ledge test) and abnormal hindlimb clasping posture compared to intact controls (Fig. 4b). We also evaluated whether ataxia-like motor impairment induced by neuroinflammation was associated with the loss of Purkinje cells, a typical neuropathologic feature of CAs^15^. Relative to control mice, LPS-injected mice exhibited > 50% reduction in calbindin protein, which indicates Purkinje cell death, in the cerebellum 7 days after LPS injection (Fig. 4c). As shown by western blotting and double immunostaining, the activation of caspase-3 showed a time-dependent increase in the LPS-injected cerebellum compared to that of intact controls (Fig. 4d; Suppl. Fig. S1), and cleaved-caspase-3 was observed in cerebellar Purkinje cells following LPS administration in vivo (Fig. 4e).

## Discussion

CAs are neurological conditions resulting from cerebellar inflammation or damage due to various causes^1,3,4^. Recent global studies on ataxia report an overall prevalence rate of 26 cases in every 100,000 children^16^. The causes of CA can be typically divided into three main categories: hereditary, idiopathic, and acquired^17^. A recent accumulation of clinical and preclinical evidence supports the hypothesis that cerebellar inflammation plays a significant role in the progression of CAs. For example, neurotoxic inflammatory responses, involving the production of inflammatory molecules by activated microglia and astrocytes, have been observed in patients and in animal models of hereditary CA, especially spinocerebellar ataxia^6,7^. In addition, some studies suggest that inflammatory responses induced by viral infection or toxic exposures (exposure to alcohol, drugs, or environmental toxins) can cause CA because the cerebellum is especially vulnerable to poisoning and intoxication^8,9,18^. These research findings suggest that the upregulation of inflammatory responses in the cerebellum may be an important mechanism in the progression of CAs and that the control of neuroinflammation may be a potential therapeutic target for the management of CAs.

The development of effective targeted therapies against CA has been hindered by its complex and wide-ranging causes^3^ and the lack of suitable animal models. Here, we evaluated the suitability of LPS as a potent promoter of inflammation in the cerebellum to develop a mouse model of ICA. We observed that mice with LPS-induced CA develop severe cerebellar inflammation associated with glial activation, resulting in the increased expression of the proinflammatory molecules IL-1β, TNFα, MCP-1, and MIP-1α (Figs. 1, 2). IL-1β and TNFα were mainly produced by activated microglia (Fig. 1) and MCP-1 and MIP-1α, which are known to possess chemoattractant activity and which increased soon after LPS treatment, were predominantly produced by microglia and astrocytes, respectively (Fig. 2). The upregulation of chemoattractants such as MCP-1 and MIP-1α following an inflammatory stimulus plays an important role in the trafficking of microglia toward the site of damage. For instance, increased expression of MCP-1 after hypoxic-ischemic injury facilitates the migration of microglia to injury sites^19^, and the upregulation of MIP-1α induces microglial accumulation in the infarcted brain^20^. Together with this previous evidence, our results suggest that the production of MCP-1 and MIP-1α following LPS treatment might contribute to glial migration and increased inflammatory responses.

In neurodegenerative disorders, there are two primary types of activated microglia with different functions: the M1 phenotype producing pro-inflammatory cytokines and the M2 phenotype producing anti-inflammatory mediators such as IL-10, arginase-1, and transforming growth factor- β, which inhibit pro-inflammatory responses, improve neuronal repair, and promote phagocytosis of abnormal proteins^21,22^. The cell surface markers and cytokine expression levels of M1- and M2-type microglia in the LPS-exposed cerebellum indicate that many microglia were activated and polarized toward an M1 phenotype after LPS exposure, demonstrated by the increases in the levels of CD86 and iNOS and by the decreases in the levels of IL-10 (Fig. 3). Consistent with the induction of the proinflammatory molecules, such as IL-1β and TNFα (Fig. 1), these results suggest that LPS administration promote a shift in activated microglia, resulting in the induction of inflammatory effects in the LPS-treated cerebellum.

Accumulating evidence demonstrates that the loss of Purkinje cells, which is a typical pathological feature of CAs, can be associated with inflammation in various types of CA^1,7,23^. Moreover, Purkinje cell loss and dysfunction are the most frequent features in patients and animal models with ataxic symptoms^7,24^. In addition to inflammatory responses of activated glia in the cerebellum, we observed the apoptotic death of Purkinje cells in the LPS-treated cerebellum, and injured mice showed evidence of motor dysfunction, such as impaired coordination, loss of balance, and abnormal hindlimb clasping postures (Fig. 4).

In conclusion, we examined the potential of an intracerebellar injection of LPS to develop a mouse model of ICA. LPS-induced ICA mice displayed impairments of balance and motor control, indicating cerebellar dysfunction, and these features were accompanied by the upregulation of proinflammatory molecules via glial activation, followed by apoptotic death of Purkinje cells (Fig. 4f). Thus, these results demonstrate that the LPS-treated mice manifest inflammatory responses and typical symptoms of patients with CA, suggesting their possible utility as an experimental animal model of ICA.

## Methods

### Animals

Male C57BL/6 mice (10-week-old; Daehan Biolink Co., Ltd, Eumseong, Korea) were housed in a controlled environment (23 ± 2 °C and 50–60% humidity) with a 12 h photoperiod and free access to food and water. All experimental animal procedures were performed in accordance with approved animal protocols and the guidelines of the Animal Care Committee of Kyungpook National University (No. KNU 2019-0002). All efforts were made to minimize both the suffering and the number of animals involved in the experiments.

### ICA animal model

To develop an ICA animal model, LPS (5 μg/5 μL) was injected directly into the mouse cerebellum as previously described but with some alterations^25^. Briefly, male C57BL/6 mice (10 week old, Daehan Biolink Co., Ltd, Eumseong, Korea) were anesthetized deeply with intraperitoneal administration of 115 mg/kg of ketamine (Yuhan, Republic of Korea) and 23 mg/kg of Rompun (Bayer Korea Ltd., Republic of Korea) and placed on a stereotaxic frame (David Kopf Instruments, Tujunga, CA, USA). For injection into the cerebellum, a midline sagittal incision was made to expose the skull, and a burr hole was drilled. A total of 5 μL of LPS (1 mg/mL) or phosphate buffered saline (PBS) was injected once in the cerebellum (AP: − 2.5 mm; DV: − 2.5 mm, relative to the lambda) at a rate of 0.5 μL/min through a syringe pump connected to a Hamilton syringe (10 μL, 30G). The needle was left in place for an additional 5 min to limit reflux along the injection tract. Following injection, mice were moved into a cage with a warming pad to recover from surgery.

### Primary glial cultures

Mixed primary glial cell cultures were prepared from 2-days-old C57BL/6 mice by chopping and mechanically disrupting the whole brain using a nylon mesh, as described previously^26^. Briefly, the cells were grown in DMEM with 10% heat-inactivated fetal bovine serum (FBS, Gibco BRL, Grand Island, NY, USA) and 1% penicillin-streptomycin (Gibco BRL, Grand Island, NY, USA) at 37 °C in a humidified atmosphere of 5% CO_2_. Culture media were replaced with fresh media after the initial 5 days and then every 3 days. Primary microglia were obtained from mixed glial cultures via mild trypsinization. After culture for 3 week, mixed glial cells were treated for 20 min with a trypsin solution (0.25% trypsin-EDTA, Gibco BRL, Paisley, Scotland) diluted 1:3 in serum-free DMEM. This resulted in the detachment of astrocytes, whereas microglia remained attached to the bottom of the culture flask. The detached astrocytes were collected by centrifugation at 5,000 × *g* for 10 min for primary astrocytes cultures and the remaining primary microglia were used for experiments. The collected microglia and astrocytes were grown and maintained in DMEM with 10% FBS and 1% penicillin-streptomycin.

### Measurement of MCP-1 and MIP-1α by traditional RT-PCR in glial cultures

To induce glial activation, microglia were treated for 6 h with final concentrations of 100 ng/mL LPS from *Escherichiacoli* 0111:B4 (Sigma, USA) diluted in DMEM with 10% FBS and 1% penicillin-streptomycin. Astrocytes were stimulated for 6 h with a combination of LPS (100 ng/mL) and IFN-γ (50 U/mL; R&D systems, Minneapolis, USA), which boosts the inflammatory response via a massive release of pro-inflammatory factors such as TNFα, IL-6 and nitric oxide^27^. The cells were washed and collected in TRIzol reagent (Life Technologies, Gaithersburg, MD) according to the manufacturer’s instructions. Reverse transcription was conducted using Superscript II (Invitrogen, Gaithersburg, MD, USA) and oligo (dT) primers. Traditional PCR amplification using specific primer sets was generated by 25–30 cycles at an annealing temperature of 55–60 °C. The specific primers were as follows; GADPH fwd: 5′-ACCACAGTCCATGCCATCAC-3′, GADPH rev: 5′-TCCACCACCCTGTTGCTGTA-3′, MIP-1α fwd: 5′-TCTGCAACCAAGTCTTCTCAG-3′, MIP-1α rev: 5′-GAAGAGTCCCTCGATGTGGCTA-3′, MCP-1 fwd: 5′-CAGCAGGTGTCCCAAAGAA-3′, MCP-1 rev: 5′-CTTGAGGTGGTTGTGGAAAAG-3′. Amplified products were electrophoresed on an agarose gel and visualized by ethidium bromide staining. GAPDH was used as the internal control.

### Behavioral tests

The rotarod testing began 1 day before the LPS injection and it was then conducted weekly for 4 week. For the simple composite phenotype scoring system described below, behavioural testing was performed once, 4 week after LPS injection.

#### Rotarod test

The rotarod test was used to evaluate motor coordination and balance according to the method described by Zhang et al.^28^. Experimental mice were carefully placed on a rotating rod weekly for 4 week. The rotational speed was linearly increased from 4 to 40 rpm, and then remained at this speed for the remaining 5 min. The total time that the mice could stay on the rotating rod was recorded. To avoid muscle fatigue, each mouse had 10 min of rest between each trial.

#### Simple composite phenotype scoring system

To quantify disease severity in the LPS-induced mouse model of CA, a previously described scoring system combining a ledge test and hindlimb clasping test was used with modifications^14^. The scoring was performed at 14 week of age. All tests were scored on a scale of 0–3 and combined into a composite phenotype score of 0–6: 0 indicates absence of the relevant phenotype, and 3 indicates the most severe symptoms. Each test was performed in triplicate.

*The ledge test* Imbalance and incoordination were scored using a ledge test. A score of 0 was applied if a mouse typically walked along the ledge without losing its balance. A score of 1 was applied if a mouse walked in an asymmetrical posture along the ledge. A score of 2 was applied if a mouse lost its footing while walking along the ledge. A score of 3 was applied if a mouse did not effectively use its hind legs.

*Hindlimb clasping test* Hindlimb clasping has been used as a marker of disease progression in CA mouse models^29^. A score of 0 was applied if the hindlimbs were consistently splayed outward, away from the abdomen. A score of 1 was applied if one hindlimb was retracted toward the abdomen for more than 50% of the time measured. A score of 2 was applied if both hindlimbs were partially retracted toward the abdomen for more than 50% of the time measured. A score of 3 was applied if the hindlimbs were entirely retracted and touching the abdomen for more than 50% of the time measured.

### Immunofluorescence staining

Mice were anesthetized with a ketamine/xylazine mixture and perfused transcardially with 4% paraformaldehyde (PFA; Sigma, St. Louis, MO, USA) in 0.1 M PBS, as described previously^30^. The brains were removed and post-fixed in 4% PFA overnight before transfer into 30% sucrose in 4% PFA for 48 h at 4 °C. Free-floating sagittal cerebellar sections (30 μm thick) were collected using a HM525 NX cryostat(Thermo Fisher Scientific, Scoresby, VIC, Austrailia), before being washed with 0.1 M PBS and then blocked with 0.5% BSA and 0.1% Triton X-100 in 0.1 M PBS for 1 h. Sections were incubated with two kinds of primary antibodies overnight at 4 °C. The following primary antibodies were used: anti-ionized calcium-binding adapter molecule 1 (Iba1, 1:2,000, Wako, Osaka, Japan), anti-glial fibrillary acidic protein (GFAP, 1:2,000, Millipore, Billerica, MA, USA), anti-tumor necrosis factor alpha (TNFα, 1:500, Abcam, Cambridge, UK), anti-interleukin 1 beta (IL-1β, 1:500, Abcam, Cambridge, MA, USA), anti-monocyte chemoattractant protein 1 (MCP-1, 1:100, Abcam, Cambridge, UK), anti-macrophage inflammatory protein 1 alpha (MIP-1α, 1:100, R&D Systems, Minneapolis, MN, USA), anti-calbindin-D-28K (Cal-D28K, 1:500, Sigma, St. Louis, MO, USA), anti-cleaved caspase-3 (1:400, Cell Signaling, Beverly, MA, USA), anti-cluster of differentiation 86 (CD86, 1:200, Invitrogen, Carlsbad, CA, USA), and anti-cluster of differentiation 206 (CD206, 1:200, R&D Systems, Minneapolis, MN, USA), followed by incubation with Texas Red- (1:400, Vector Laboratories, Burlingame, CA) and FITC-labeled IgG antibody (1:400, Jackson ImmunoResearch, West Grove, PA), and counterstained with DAPI (Sigma). Slides were mounted with Vectashield mounting medium (Vector Laboratories, Burlingame, CA). The stained sections were examined under a microscope (Axio Imager; Carl Zeiss, Gottingen, Germany).

### Western blot analysis

Total cell lysates from the vermis of the mouse cerebellum were prepared for Western blotting as previously described^31^. The cerebellar vermis were separated, and each tissue was homogenized in lysis buffer (58 mM Tris-HCl, pH 6.8; 10% glycerol; and 2% SDS) with a protease inhibitor cocktail (1:100, Millipore, Burlington, MA, USA) and phosphatase inhibitor cocktail (1:100, Cell Signaling Technology). The lysates were cleared by centrifugation, and protein concentration was determined using a BCA kit (Bio-Rad Laboratories, Hercules, CA, USA). Proteins were separated using gel electrophoresis and transferred onto PVDF membranes using an electrophoretic transfer system (Bio-Rad Laboratories). Membranes were incubated overnight at 4 °C with the following primary antibodies: anti-ionized calcium-binding adapter molecule 1 (Iba1, 1:1,500, Wako, Osaka, Japan), anti-glial fibrillary acidic protein (GFAP, 1:2,000, Millipore, Billerica, MA, USA), anti-tumor necrosis factor alpha (TNFα, 1:1,000, Abcam, Cambridge, UK), anti-interleukin 1 beta (IL-1β, 1:1,000, Abcam, Cambridge, MA, USA), anti-monocyte chemoattractant protein 1 (MCP-1, 1:500, Abcam, Cambridge, UK), anti-macrophage inflammatory protein 1 alpha (MIP-1α, 1:1,000, R&D Systems, Minneapolis, MN, USA), anti-calbindin-D-28 K (Cal-D28K, 1:2,000, Sigma, St. Louis, MO, USA), anti-cleaved caspase-3 (1:1,000, Cell Signaling, Beverly, MA, USA), anti-caspase-3 (1:1,000, Cell Signaling, Beverly, MA, USA), anti-cluster of differentiation 86 (CD86, 1:1,000, Invitrogen, Carlsbad, CA, USA), anti-cluster of differentiation 206 (CD206, 1:1,000, R&D Systems, Minneapolis, MN, USA), anti-inducible nitric oxide synthase (iNOS, 1:1,000, Abcam, Cambridge, UK), anti-interleukin 10 (IL-10, 1:1,000, Abcam, Cambridge, MA, USA), and anti-β-actin (1:2,000, Santa Cruz, CA, USA). After incubation in horseradish peroxidase (HRP)-conjugated secondary antibodies (Amersham Biosciences, Piscataway, NJ, USA), blots were developed using enhanced chemiluminescence western blotting detection reagents (Amersham Biosciences). For semi-quantitative analyses, band densities were measured by an ImageQuant LAS 500 imager (GE Healthcare Life Science). The intensity of each protein band was calculated using Multi Gauge V3.0 software and normalised to that of the corresponding β-actin band. The images of the western bands in figures are representative examples. The fuller-length blots of all images are shown in Supplementary Fig. 2. All of the antibodies used in this study were described in Suppl. Table S1.

### Statistical analysis

All statistical analyses were conducted using SigmaPlot software, version 12.0 (Systat Software, San Leandro, CA). Data are depicted as mean ± standard deviation (SD). Statistical significance was determined using one-way analysis of variance (ANOVA) followed by Tukey’s post hoc test for multiple comparisons among the groups or, in some cases, Mann–Whitney U statistic and two-sided paired t-test to compare the two groups.

## Supplementary information

Supplementary Information.
