# Supplementary material for: Lipopolysaccharide administration for a mouse model of cerebellar ataxia with neuroinflammation
Source: Sci Rep. 2020 Aug 7;10:13337. doi: 10.1038/s41598-020-70390-7 (PMC7414878; doi:10.1038/s41598-020-70390-7)
Supplement: Supplementary file 1 — Supplementary Information. [file 41598_2020_70390_MOESM1_ESM.docx]

Supplementary information

**Lipopolysaccharide administration for a mouse model of cerebellar ataxia with neuroinflammation**

Jungwan Hong, Dongyeong Yoon, Youngpyo Nam, Donggun Seo, Jong-Heon Kim, Min Sung Kim, Tae Yong Lee, Kyung Suk Kim, Pan-Woo Ko, Ho-Won Lee, Kyoungho Suk, Sang Ryong Kim

Supplementary information:

Supplementary Table 1

Supplementary Figure 1

Supplementary Figure 2

**Table S1. List of antibody**

| **Antibody** | **Host** | **Dilution (IF)** | **Dilution (WB)** | **Supplier** | **Catalog #** |
| --- | --- | --- | --- | --- | --- |
| Iba-1 | Rabbit | 1:2000 | 1:1500 | Wako | 019-19741 |
|  | Goat | 1:1000 |  | Abcam | ab5076 |
| GFAP | Mouse | 1:2000 | 1:2000 | Milipore | MAB360 |
| TNFα | Rabbit | 1:500 | 1:1000 | Abcam | ab6671 |
| IL-1β | Rabbit | 1:500 | 1:1000 | Abcam | ab9722 |
| MCP-1 | Rabbit | 1:100 | 1:500 | Abcam | ab8101 |
| MIP-1α | Rabbit | 1:100 | 1:1000 | R&D System | MAB4501 |
| Calbindin | Mouse | 1:500 | 1:2000 | Sigma | C9848 |
| C-Cas-3 | Rabbit | 1:400 | 1:1000 | Cell Signaling | CST9664 |
| Cas-3 | Rabbit |  | 1:1000 | Cell Signaling | CST9662 |
| CD86 | Rat | 1:200 | 1:1000 | Invitrogen | 14-0862-81 |
| CD206 | Mouse | 1:200 | 1:1000 | R&D System | AF2535 |
| iNOS | Rabbit |  | 1:1000 | Abcam | ab3523 |
| IL-10 | Rabbit |  | 1:1000 | Abcam | ab9969 |
| β-actin | Mouse |  | 1:2000 | Santa Cruz | sc-47778 |
| Anti-Rabbit FITC | Donkey | 1:400 |  | Jackson ImmunoResearch | 711-095-152 |
| Anti-Mouse FITC | Horse | 1:400 |  | Vector Laboratories | FI-2000 |
| Anti-Goat FITC | Rabbit | 1:400 |  | Vector Laboratories | FI-5000 |
| Anti-Rabbit TexasRed | Goat | 1:400 |  | Vector Laboratories | TI-1000 |
| Anti-Rat alexa 594 | Goat | 1:400 |  | Abcam | ab150160 |
| Anti-Mouse TexasRed | Horse | 1:400 |  | VectorLab | TI-2000 |
| Anti-Mouse HRP | Rabbit |  | 1:4000 | Amersham Biosciences | 61-6520 |
| Anti-Rabbit HRP | Goat |  | 1:4000 | Amersham Biosciences | 65-6120 |
| Anti-Rat HRP | Goat |  | 1:4000 | Milipore | AP183P |


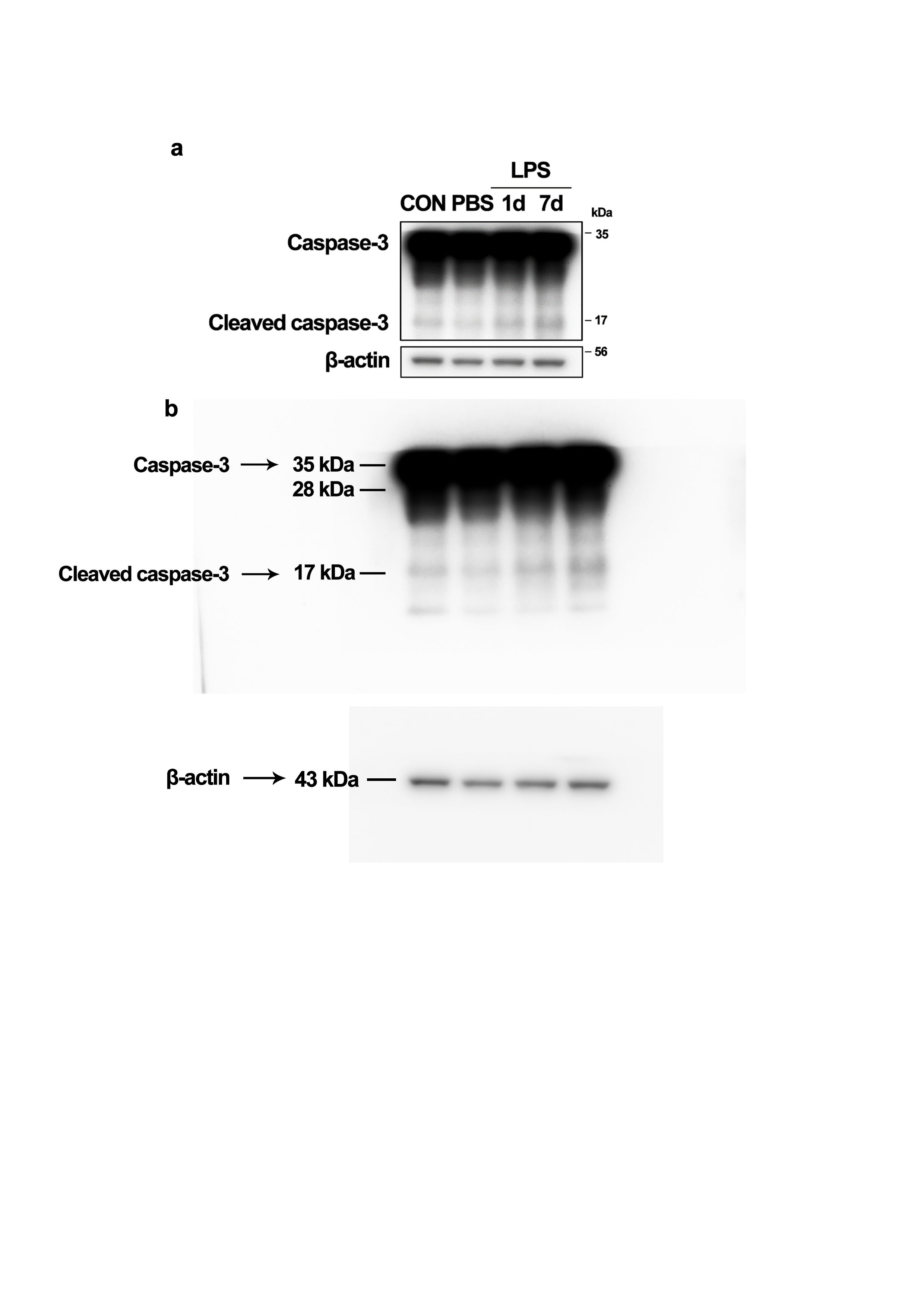


**Figure S1. The expression levels of full-size and cleaved caspase-3 on a single membrane following LPS administration.**

(**a**) Western blotting using anti-caspase-3 (Cell Signaling; Cat. #­ CST 9662) showed the increases in the protein levels of cleaved caspase-3 1 d and 7 d after LPS injection compared to controls. (**b**) Full-length images of the cropped images shown in the upper panels.


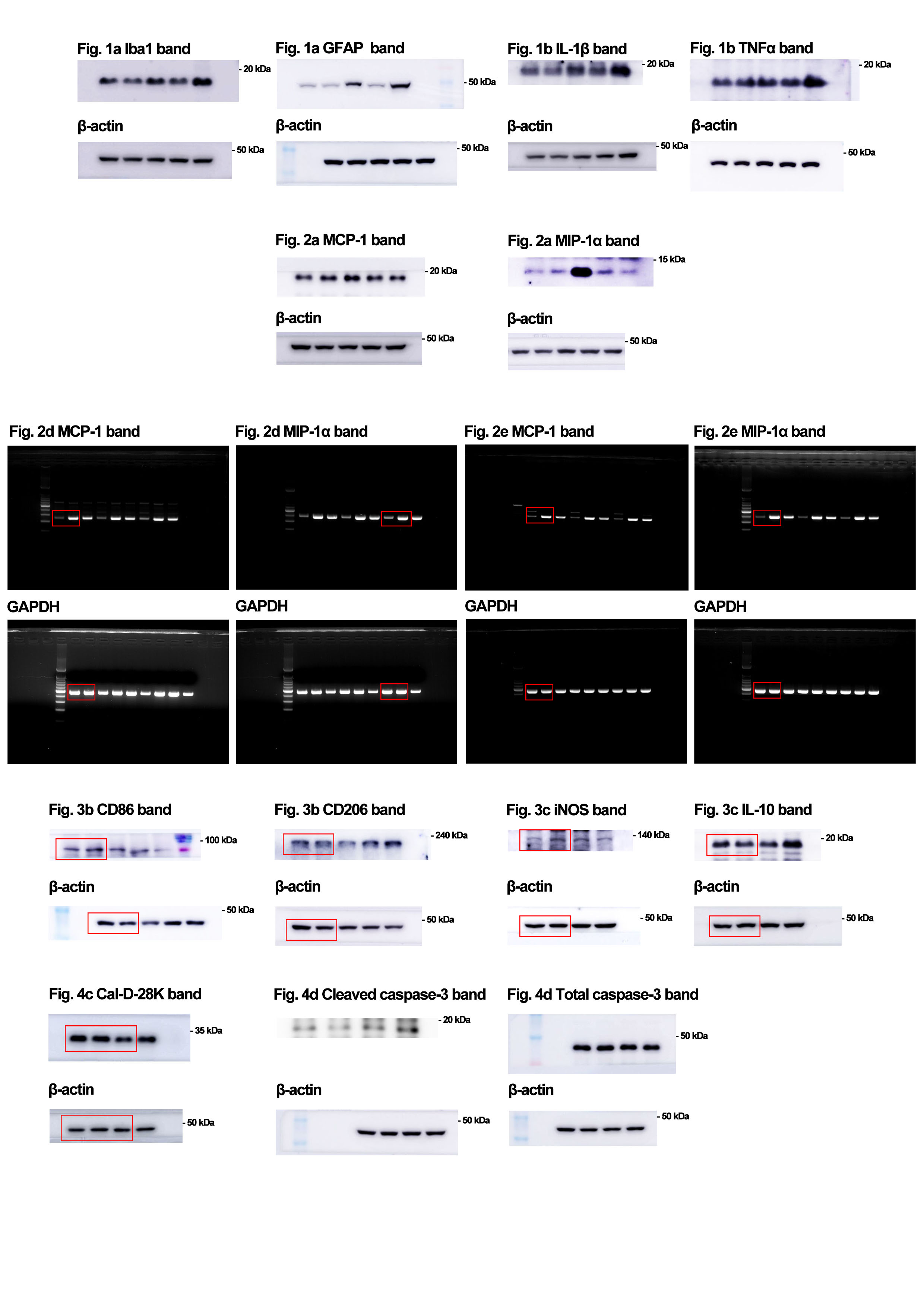


**Figure S2. The original blots/gels of cropped images shown in the main figures.**
